# The combination therapy with EpCAM/CD3 BsAb and MUC-1/CD3 BsAb elicited antitumor immunity by T-cell adoptive immunotherapy in lung cancer

**DOI:** 10.7150/ijms.61681

**Published:** 2021-07-31

**Authors:** Ce Wang, Shang Chen, Yingjuan Wu, Di Wu, Jingbo Wang, Furong Li

**Affiliations:** Shenzhen key laboratory of stem cell research and clinical transformation, Guangdong Engineering Technology Research Center of Stem cell and Cell therapy, Translational Medicine Collaborative Innovation Center, The Second Clinical Medical College (Shenzhen People's Hospital), Jinan University, Shenzhen 518020, China.

**Keywords:** non-small cell lung cancer, EpCAM/CD3 BsAb, MUC-1/CD3 BsAb, immunotherapy

## Abstract

Lung cancer remains a global challenge due to high morbidity and mortality rates and poor response to treatment, and there are still no effective strategies to solve it. The bispecific antibody (BsAb) is a novel antibody, which can target two different antigens and mediate specific killing effects by selectively redirecting effector cells to the target cells. In this study, we combined two BsAbs to achieve a dual-target therapy strategy of EpCAM^+^ and MUC-1^+^ with high affinity and specificity. The results showed that the combination of two BsAbs against EpCAM and MUC-1 could inhibit the growth of lung cancer more effectively in cell lines and primary tumors. The superior antitumor effect of two BsAbs could be attributable to enhanced CTL and increased production of type I IFNs. At the same time, the combination of EpCAM/CD3 BsAb and MUC-1/CD3 BsAb significantly regulated T population in the TDLNs. Therefore, we have found a potential immunotherapeutic strategy, which was the combination therapy with EpCAM/CD3 BsAb and MUC-1/CD3 BsAb for the treatment of non-small cell lung cancer.

## Introduction

Lung cancer was one of the most common malignant tumors in the world 1, whose morbidity and mortality were the highest among all the malignant tumors. Of all the lung cancer patients, 80%-85% suffered from non-small cell lung cancer (NSCLC). In recent years, the incidence of NSCLC was younger and rising worldwide. Although the clinical diagnosis and treatment level was developing rapidly, the 5-year survival rate of NSCLC is only 15% 2-3. Therefore, there was an urgent need to study the feasibility of anti-lung cancer treatment.

Because BsAbs could target two different antigens, they were widely used in the field of tumor therapy. In the past decade, the research of BsAb has been growing steadily. Immune cells played an important role in the immunotherapy of tumor. Mature T cells marked with CD3 played an important role in the immune response and became the preferred target cells for the study of effect of BsAb 4. At present, catumaxomab, which was approved by EU in 2009, was the first BsAb targeted at EpCAM/CD3 for cancer treatment 5. The anti-tumor effect of catumaxomab was achieved by recruiting T cells to kill target cells, using antibody dependent cell-mediated cytotoxicity and cytokine-mediated cytotoxicity 6. In addition, the clinical trials for ovarian cancer, gastric cancer, non-small cell lung cancer and breast cancer were also in progress 7. Another commercially available monoclonal antibody was blinatumomab, which could specifically bind CD3^+^ T lymphocytes and CD19^+^ B lymphocytes to mediate high level cytotoxic effect of T cells on lymphoma cells 8. The results showed that BsAbs have obvious advantages. They had play an effective killing effect at very low concentration of antibody drugs. However, tumors were usually pathological changes caused by various reasons, regulated by multiple signal pathways, and having more than one surface specific antigen. As a result, blocking multiple targets at the same time might achieve better therapeutic effect 9. Therefore, we combined two BsAbs for anti-tumor treatment. In addition, it was still a problem to be solved to determine the best time and treatment course for the use of antibody drugs.

As we all know, epithelial cell adhesion molecule (EpCAM) was a single transmembrane protein of adhesion molecule family. It was highly expressed in the basement membrane of many kinds of malignant tumors, and abnormally expressed in many kinds of human tumor tissues such as colorectal cancer, prostate cancer and gastric cancer. It participated in the process of proliferation, differentiation, adhesion, invasion and migration of tumor cells by regulating related signal pathways or target genes. It was closely related to the occurrence and development of tumors 10-12. It has been reported that high expression of EpCAM was closely related to lung cancer metastasis and poor prognosis, thus might play an important role in the occurrence and development of lung cancer 13. In addition, Pak et al. found that the positive expression rate of EpCAM protein in tumor tissues of NSCLC patients in stage II-IV was 73.33%, significantly higher than 47.06% in stage I of NSCLC patients 14.

In addition, polymorphic epithelial mucin (MUC-1) was a kind of high molecular weight glycoprotein, which had abnormal expression in epithelial cell-derived malignant tumors and played an important role in tumor growth, invasion, development and metastasis 15. Giatromanelaki et al. found that in NSCLC, the expression of MUC-1 was an important prognostic factor independent of T and N stages, which was related to the expression of vascular endothelial growth factor (VEGF), which leads to angiogenesis and tumor migration 16. The expression of MUC-1 in lung cancer could provide effective information for clinical judgment of lymph node metastasis trend, prediction of lymph node metastasis potential and judgment of the prognosis of NSCLC, and played a guiding role in prognosis and postoperative treatment of NSCLC 17.

In this study, we combined EpCAM/CD3 BsAb and MUC-1/CD3 BsAb to target both EpCAM and MUC-1 on the surface of tumor cells. The results showed that the combination of two BsAbs could effectively inhibit the growth of NSCLC and promote the apoptosis of tumor cells. We further confirmed that the combination of BsAbs therapy could effectively inhibit tumor growth and inflammatory response *in vivo*, suggesting its immunotherapeutic potential in solid tumor therapy *in vivo*.

## Methods

### Materials

Human blood buffy coats from healthy donors were obtained from Shenzhen Blood Center (Shenzhen, China), and tested negative for blood-born pathogens using the standard protocols of the blood center. All of the protocols involving human blood samples were approved by the Institutional Review Board of the Shenzhen Blood Center. 5-6 weeks old female NOD/SCID mice were purchased from Gem Pharmatech LLC (Jiangsu, China), 5-6 weeks old female BALB/c mice were purchased from Guangdong Province Laboratory Animal Center (Guangzhou, China) and maintained in the institutional animal care facility. All animal protocols were approved by Institutional Animal Care and Usage Committee of Shenzhen People's Hospital.

### Sample Collection

5 patients with primary lung cancer from January 2016 to June 2019 in Shenzhen People's Hospital. Informed consent for the additional core-needle biopsy and experimental use of tumor samples was obtained from all patients, following a protocol approved by the Ethics Committee of Shenzhen People's Hospital.

### The EpCAM/CD3 BsAb and the MUC-1/CD3 BsAb

The EpCAM/CD3 BsAb and the MUC-1/CD3 BsAb in this study were obtained from BenHealth Biopharmaceutical (Shenzhen) Co. LTD. The EpCAM/CD3 BsAb or the MUC-1/CD3 BsAb was connected MUC1 antibody or EpCAM antibody and CD3 antibody by biodegradable nanomaterials. Briefly, MUC1/CD3 BsAb or EpCAM/CD3 BsAb were obtained by using 1-Ethyl-3-(3-dimethylaminopropyl) carbodiimide (EDC.HCl) and N-hydroxysuccinimide (NHS) to bind MUC1 antibody or EpCAM antibody and CD3 antibody to polylactic acid glycolic acid (PLGA), respectively.

### Cell lines and cell culture conditions

A549, H466 and H1975 cells were obtained from Shanghai Institutes for Biological Sciences Cell Bank (Shanghai, China), and maintained in Dulbecco's Modified Eagle Medium (DMEM high glucose, Invitrogen, CA, USA) supplemented with 10% fetal bovine serum (FBS, Thermo Scientific, MA, USA). All cells were incubated at a 37 °C humidified incubator containing 5% CO_2_.

### T cells culture, stimulation and activation

Human monocytes were enriched by plastic adherence of peripheral blood mononuclear cells (PBMCs) in a 100 mm dish at 37 °C, 5% CO_2_. After 2 h of incubation, the nonadherent cells were removed. The activated T cells (aT cells) were harvested after cultured with anti-human CD3 antibody (1 μg/ml) and anti-human CD28 antibody (1 μg/ml) followed by IL-2 (100 IU/ml) for 72 h. And then the aT cells were treated with or without EpCAM/CD3 BsAb (10 μg/ml) or MUC-1/CD3 BsAb (10 μg/ml) followed by IL-2 (100 IU/ml) for 72 h, and then washed twice by PBS to acquire different BsAb treated aT cells (Med: medium, EpCAM: EpCAM/CD3 BsAb, MUC-1: MUC-1/CD3 BsAb and EpCAM & MUC-1: EpCAM/CD3 BsAb & MUC-1/CD3 BsAb).

### The binging efficiency of T cells and tumor cells

A549, H466 and H1975 cells were labeled with Dil (2 μg/ml). The different BsAb treated T cells were labeled with Calcein-AM (2 μM). The activated T cells and target cells (A549, H466 and H1975) were cultured in 24 well plates with effect/target (E: T) ratio of 10: 1 for 6 h, and then the cells were harvested. The binging efficiency of cells was detected by flow cytometry.

### Cytokines ELISA assay

A549, H466 and H1975 cells or primary tumor cells were exposed to different BsAb treated T cells for 72 h. The supernatant of the culture medium was collected. The levels of IL-6 and IFN-γ in the medium were determined using ELISA kit (eBioscience, San Diego, CA) according to the manufacturer's instructions.

### Cytotoxic T-lymphocyte (CTL) response

The different BsAb treated T cells and target cells (A549, H466 and H1975 cells) were cultured in 96-well plates at various effector/target (E: T) ratios for 6 h. The LDH activity in supernatants was quantified using Cytotox96 Non-Radioactive Cytotoxicity Assay Kit (Promega, WI, USA) according to manufacturer's instruction.

Another part of H1975 cells were labeled with Dil (2 μg/ml). The different BsAb treated T cells were labeled with Calcein-AM (2 μM). T cells and target cells (H1975) were cultured in 24 well plates with effect/target (E: T) ratio of 10:1 for 24 h. CTL response was dynamically monitored by living cell workstation.

### Tumor implantation and animal immunization

5-6 weeks old female NOD/SCID mice were subcutaneously (s.c.) injected with A549, H466 or H1975 cells (5×10^6^ cells/ mouse) on right buttock. On day 7 after tumor cells implantation, mice were i.v. injected with PBS, activated T cells, or different BsAb treated activated T cells once a week for 3 weeks. Tumor diameters were measured in two dimensions every three days using a caliper, and the tumor volume was calculated according to the following formula: volume (mm^3^) = (width)^2^ × (length) × 1/2.

### Statistical analysis

Data are reported as the mean SE. The differences between the control and experimental groups were assessed using a Student's t test, and the differences among the multiple groups were analyzed using one-way ANONA (Graphpad Prism, GraphPad Software, La Jolla, CA). A value of p < 0.05 was considered statistically significant.

## Results and Discussion

### The expression of EpCAM and MUC-1 by tumor cells

**Figure 1:**
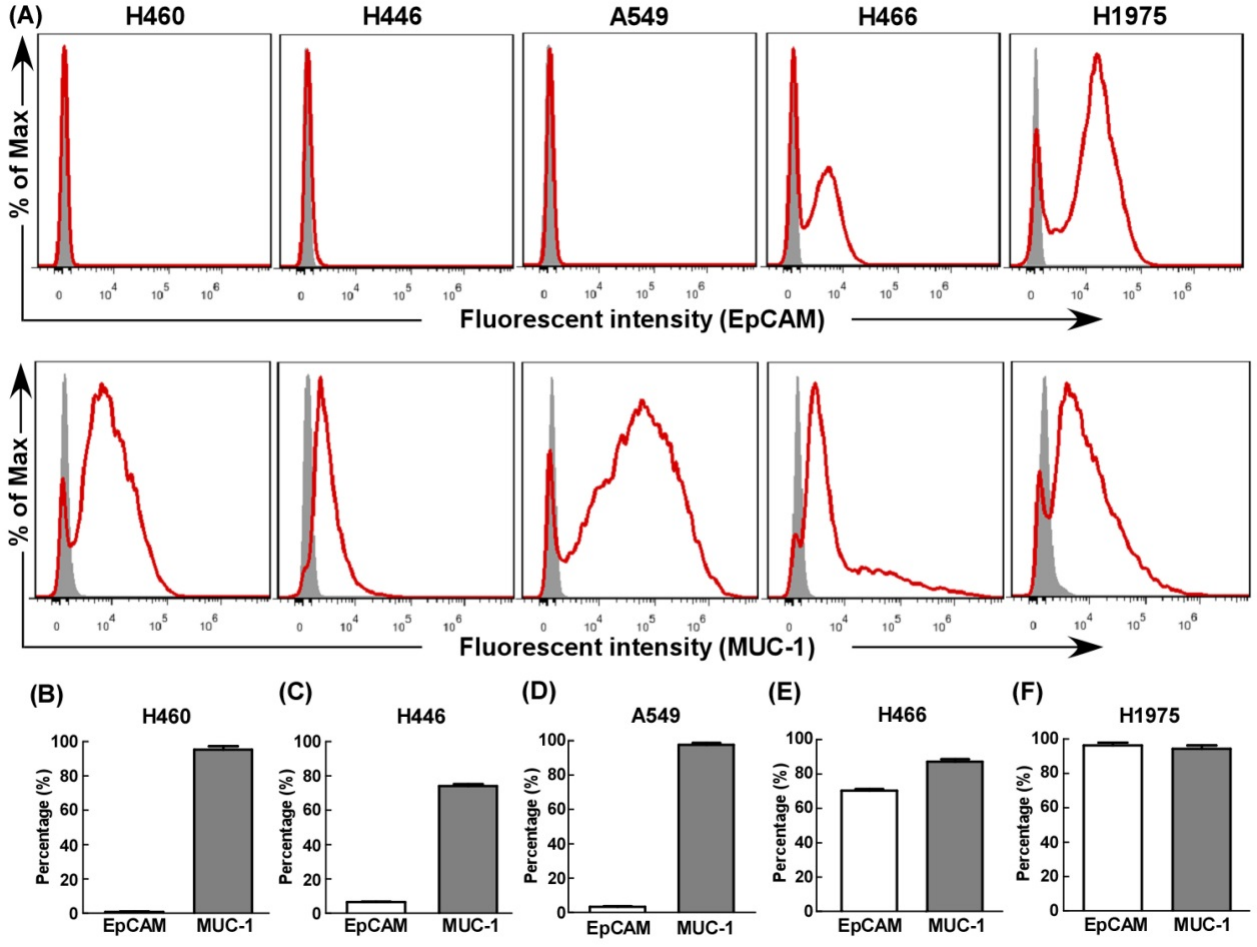
The expression of EpCAM and MUC-1 in lung tumor cell lines. The expression of EpCAM and MUC-1 were measured using flow cytometry in H460 (B), H446 (C), A549 (D), H466 (E) and H1975 (F). Experiments were repeated three times in triplicate each time (n= 3).

First, we evaluated EpCAM and MUC-1 expression in five human lung cancer cell lines using flow cytometry. All cell lines expressed MUC-1 at relative high levels (Fig. 1), but only H466 (Fig. 1E) and H1975 (Fig. 1F) expressed EpCAM at relative high levels. Hence, H466 cells and H1975 cells were selected as experimental groups in this study, both expressed relative high levels of EpCAM and MUC-1, and A549 cells were selected as control group.

### The effects of EpCAM/CD3 BsAb and MUC-1/CD3 BsAb on the binding efficiency of T cells and tumor cells

**Figure 2:**
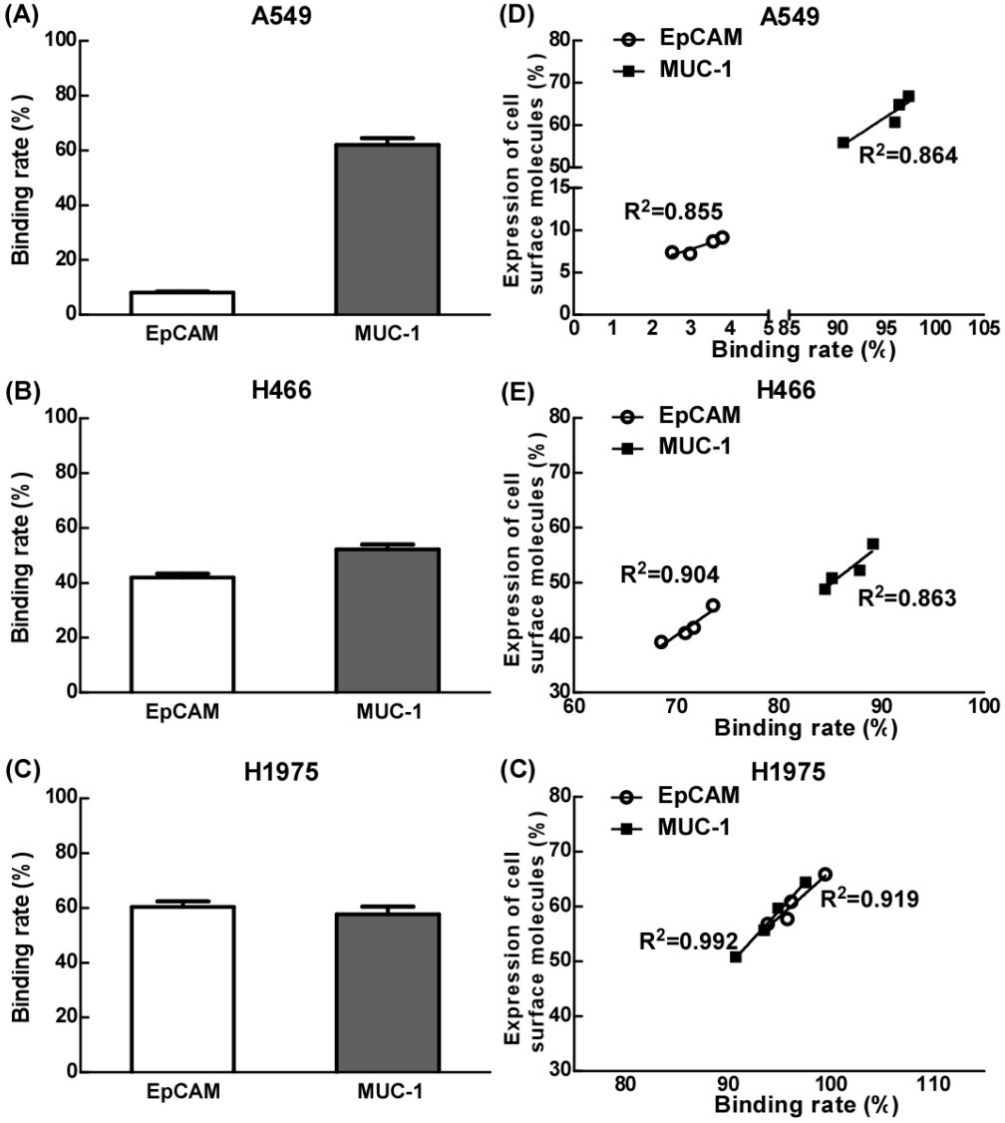
The effect of EpCAM/CD3 BsAb complexed with MUC-1/CD3 BsAb on the binding efficiency of T cells and tumor cells. A549, H466 and H1975 cells were labeled with Dil (2 µg/ml). The different BsAbs treated T cells were labeled with Calcein-AM (2 µM). The binding rate of T cells (green) and tumor cells (red) by double positive cells was recorded using quantified using flowcytometry (A-C). The correlation between binding rate (X-axis) and expression of cell surface molecules rate (Y-axis) in A549 (D), H466 (E) and H1975 (F). Bars shown are mean ± SE (n=3-4).

Our results showed that the binding efficiency of treated T cells by EpCAM/CD3 BsAb alone to A549, H466 or H1975 was 7.8%, 41.9% and 60.3% respectively, and the binding efficiency of treated T cells by MUC-1/CD3 BsAb alone to A549, H466 or H1975 was 62.0%, 52.2% and 57.6% respectively. Remarkably, the binding efficiency was positively correlated with of the expression of EpCAM or MUC-1 in tumor cells (Fig. 2D-F).

### The anti-cancer effect of combination of EpCAM/CD3 BsAb and MUC-1/CD3 BsAb in lung cell lines

**Figure 3:**
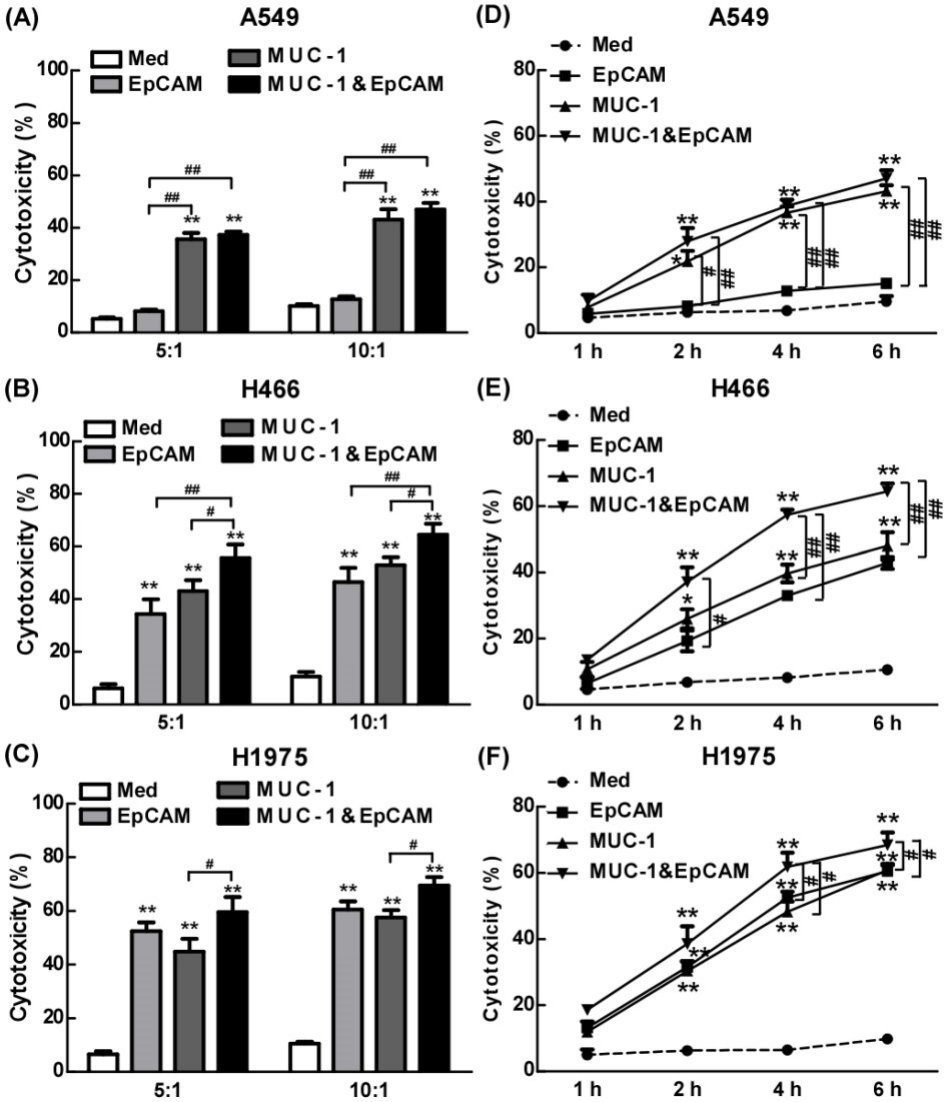
The effect of EpCAM/CD3 BsAb complexed with MUC-1/CD3 BsAb on cytotoxic T-lymphocyte (CTL) response in lung tumor cell lines. *In vitro* CTL responses were analyzed using non-radioactive cytotoxicity assay in the different BsAbs treated T cells and A549 (A), H466 (B) and H1975 (C) at various effector/target (E: T) ratios. *In vitro* CTL responses were analyzed using non-radioactive cytotoxicity assay in the different BsAbs treated T cells and A549 (D), H466 (E) and H1975 (F) at different times. Bars shown are mean ± SE (n=3-4), and differences between medium and other groups are determined using one-way ANOVA analysis. *: p < 0.05; **: p < 0.01. Differences between two different groups are statistically different, #: p < 0.05; ##: p < 0.01.

We have combined two kinds of BsAbs targeting both EpCAM and MUC-1 on the surface of lung tumors cell lines. Herein, the anti-cancer effects of the combination of EpCAM/CD3 BsAb and MUC-1/CD3 BsAb were evaluated *in vitro*. When target/effect ratio to 10:1, EpCAM/CD3 BsAb alone or MUC-1/CD3 BsAb alone significantly enhance CTL activity in H466 cells and H1975 cells, which was similar to target/effect ratio to 5:1 (Fig. 3B-C). Notably, combination of EpCAM/CD3 BsAb and MUC-1/CD3 BsAb appeared to be more potent than that by EpCAM/CD3 BsAb or MUC-1/CD3 BsAb alone to CTL activity (Fig. 3B-C). More interestingly, EpCAM/CD3 BsAb alone could not effectively enhance CTL activity, however, MUC-1/CD3 BsAb alone or combination of EpCAM/CD3 BsAb and MUC-1/CD3 BsAb could enhance CTL activity (Fig. 3A) in A549. The results might be due to the fact that the expressions of EpCAM at low level in A549 (Fig. 1), previous study confirmed that some non-small cell lung cancer (NSCLC) tumor cells had a lower expression of EpCAM 18. Therefore, it was difficult to effectively antitumor therapy with EpCAM/CD3 BsAb for EpCAM low expression of tumor. These results further suggested more effective CTL has been activated by using the combination of EpCAM/CD3 BsAb and MUC-1/CD3 BsAb than EpCAM/CD3 BsAb or MUC-1/CD3 BsAb alone.

In the present study, we dynamically observed anti-cancer effect of combination of EpCAM/CD3 BsAb and MUC-1/CD3 BsAb in H1975 through the living cell workstation. H1975 were labeled with Dil as red fluorescence, while different BsAb treated T cells were labeled with Calcein-AM as green fluorescence. The results showed that the green^+^ T cells directionally moved towards the red^+^ H1975 and continued to adsorb around the red^+^ H1975 until the red fluorescence gradually disappeared (Supplementary Video S1), confirming CTL response induced by combination of EpCAM/CD3 BsAb and MUC-1/CD3 BsAb. Hence, as a result difference of surface molecular of different cancer cell lines (Fig. 1), the combination of two BsAbs could increase the killing effect of tumor, thereby contributing to anti-cancer therapy.

To further confirm the anti-tumor effect of BsAb, we detected the CTL response from 1 h to 6 h. The results showed that the CTL response increased significantly with the increased treatment time of BsAb. It was worth noting that the BsAb has produced a significant CTL response at 2 hours. Therefore, tumor specific CTL response played an important role in the anti-tumor effect of the combination therapy with EpCAM/CD3 BsAb and MUC-1/CD3 BsAb.

### Proinflammatory effects of combination of EpCAM/CD3 BsAb and MUC-1/CD3 BsAb in lung cell lines

**Figure 4:**
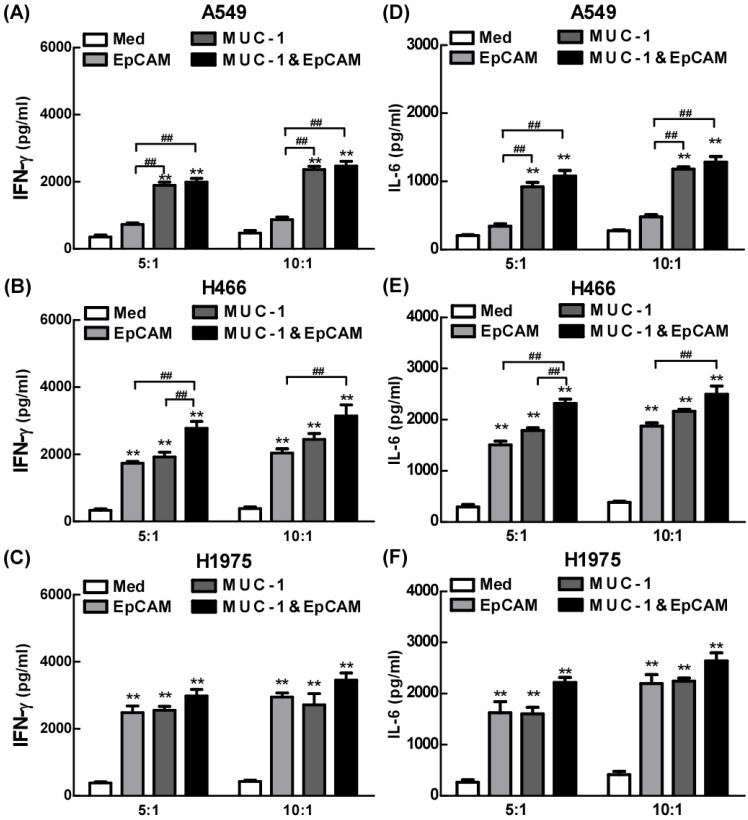
The proinflammatory effects of EpCAM/CD3 BsAb complexed with MUC-1/CD3 BsAb in lung tumor cell lines. The productions of IFN-γ (A-C) and IL-6 (D-F) in culture supernatants were measured using ELISA. Bars shown are mean ± SE (n=3-4), and differences between medium and other groups are analyzed using one-way ANOVA analysis. **: p < 0.01. Differences between two different groups are statistically different, ##: p < 0.01.

The effects of combination of EpCAM/CD3 BsAb and MUC-1/CD3 BsAb on cytokine production were further confirmed using ELISA in lung cell lines. As shown in Fig. 4, both EpCAM/CD3 BsAb alone and MUC-1/CD3 BsAb alone markedly increased the production of IFN-γ (Fig. 4B-C) and IL-6 (Fig. 4E-F), suggesting that there was no significant difference between the two treatments in H466 and H1975. However, when the target/effect ratio to 5:1 or 10:1, MUC-1/CD3 BsAb alone markedly increased the production of IFN-γ (Fig. 4A) and IL-6 (Fig. 4D), EpCAM/CD3 BsAb alone hardly up-regulated IFN-γ (Fig. 4A) and IL-6 (Fig. 4D), which might be a result of the low expression of EpCAM in A549 cells. Notably, combination of EpCAM/CD3 BsAb and MUC-1/CD3 BsAb appeared to be more potent than that by EpCAM/CD3 BsAb or MUC-1/CD3 BsAb alone to elevate the production of IFN-γ (Fig. 4A-C) and IL-6 (Fig. 4D-F) in A549, H466 and H1975. Overall, combination of EpCAM/CD3 BsAb and MUC-1/CD3 BsAb induced stronger cytokine production in A549, H466 and H1975. Besides, the proinflammatory effects of BsAbs on cancer cells appeared to be associated with the expression of specific antibodies on the surface of cancer cells, and there was a dose dependence.

### The expression of EpCAM and MUC-1 by primary tumor cells

**Figure 5:**
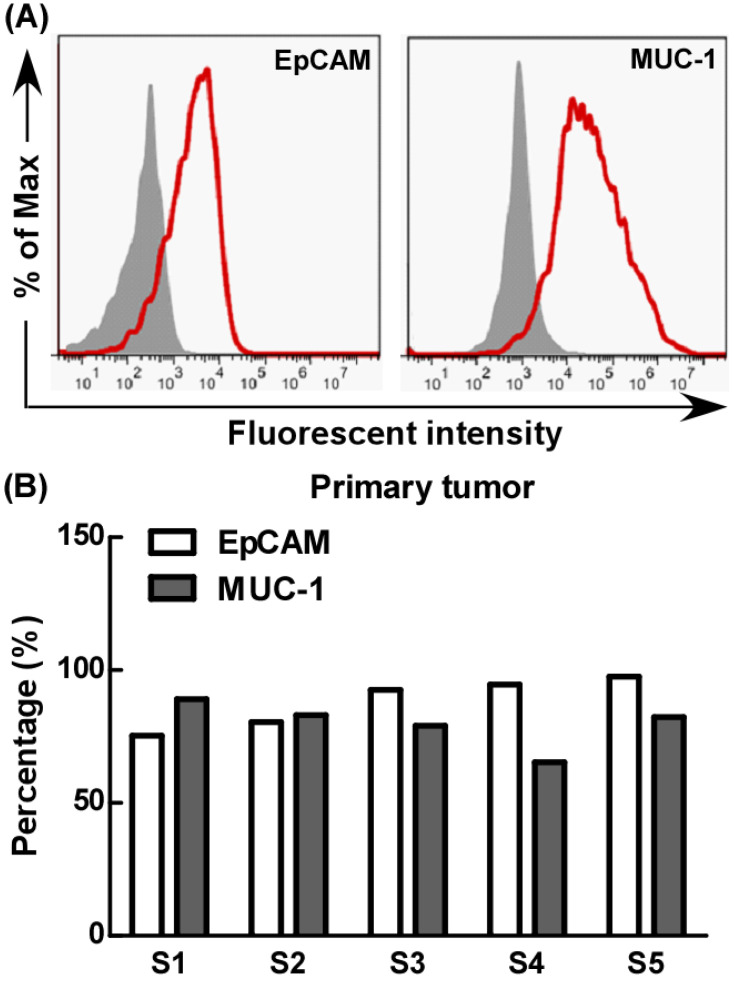
The expression of EpCAM and MUC-1 in primary tumor. Experiments were repeated five times in triplicate each time (n= 5).

Further, we evaluated EpCAM and MUC-1 expression by primary tumor cells using flow cytometry. Our results showed that the primary tumor cells expressed MUC-1 and EpCAM at relatively high levels, but there were significant differences among different primary tumors, the expression rates of MUC-1 and EpCAM were 65%-89% and 75%-97% respectively (Fig. 5).

### The anti-cancer effect of combination of EpCAM/CD3 BsAb and MUC-1/CD3 BsAb in primary tumor cells

**Figure 6:**
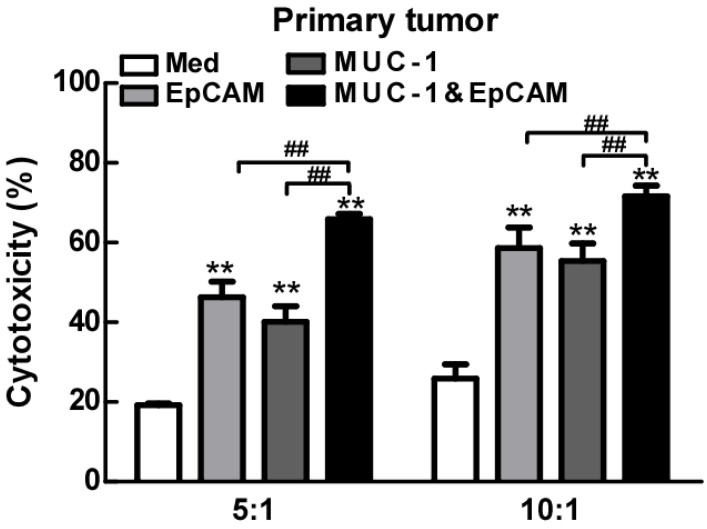
The effect of EpCAM/CD3 BsAb complexed with MUC-1/CD3 BsAb on cytotoxic T-lymphocyte (CTL) response in primary tumor. *In vitro* CTL responses were analyzed using non-radioactive cytotoxicity assay in A549 (A), H466 (B) and H1975 (C). Bars shown are mean ± SE (n=3-4), and differences between medium and other groups are determined using one-way ANOVA analysis. **: p < 0.01. Differences between two different groups are statistically different, ##: p < 0.01.

Then we investigated the anti-cancer effect of the combination of EpCAM/CD3 BsAb and MUC-1/CD3 BsAb in primary lung tumors cells. As shown in Fig. 5, when target/effect ratio was 10:1, both EpCAM/CD3 BsAb alone and MUC-1/CD3 BsAb alone significantly enhance CTL activity in primary lung tumors cells, combination of EpCAM/CD3 BsAb and MUC-1/CD3 BsAb appeared to be more potent to CTL activity. Similarly, when target/effect ratio was 5:1, combination of EpCAM/CD3 BsAb and MUC-1/CD3 BsAb appeared to be more potent to CTL activity in primary lung tumors cells (Fig. 6). These results confirmed that the combination therapy of EpCAM/CD3 BsAb and MUC-1/CD3 BsAb has more effectively induced anti-tumor CTL response in primary tumor cells. In the present study, due to different expression levels of surface molecular in primary tumor cells (Fig. 5), combination of EpCAM/CD3 BsAb and MUC-1/CD3 BsAb could increase the killing effect of tumor, thereby contributing to anti-cancer therapy.

### Proinflammatory effects of combination of EpCAM/CD3 BsAb and MUC-1/CD3 BsAb in primary tumor cells

**Figure 7:**
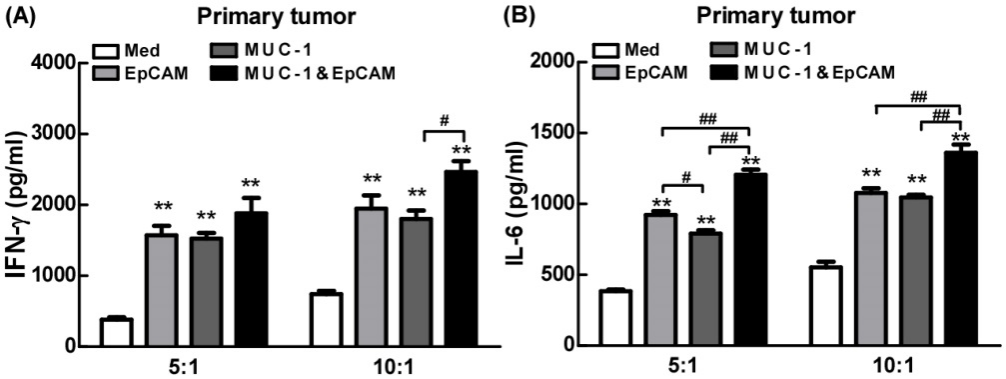
Proinflammatory effects of EpCAM/CD3 BsAb complexed with MUC-1/CD3 BsAb in primary tumor. The productions of IFN-γ (A) and IL-6 (B) in culture supernatants were measured using ELISA. Bars shown are mean ± SE (n= 5), and differences between medium and other groups are analyzed using one-way ANOVA analysis. **: p < 0.01. Differences between two different groups are statistically different, #: p < 0.05; ##: p < 0.01.

Next, the effects of combination of EpCAM/CD3 BsAb and MUC-1/CD3 BsAb on cytokine production were further confirmed using ELISA in primary tumor cells. Our results showed that combination of EpCAM/CD3 BsAb and MUC-1/CD3 BsAb significantly elevated the production of IFN-γ by 4-5 folds than untreated T cells in primary tumor (Fig. 7), which was a key cytokine indispensable to anti-tumor therapy and enhanced tumor-specific CTL responses, consistent with our previous observation 19. In addition, lack of IFN-γ production by CD8^+^ T cells suggested that the CD8^+^ T cell-mediated CTL response could be limited 20. It was noteworthy that combination of EpCAM/CD3 BsAb and MUC-1/CD3 BsAb more effectively elevated the production of IFN-γ than EpCAM/CD3 BsAb or MUC-1/CD3 BsAb alone (Fig. 7A), suggesting that the proinflammatory effects of BsAbs be associated with the expression of specific antibodies on the surface of primary tumor cells. In addition, combination of EpCAM/CD3 BsAb and MUC-1/CD3 BsAb more effectively elevated the production of IL-6 than EpCAM/CD3 BsAb alone (Fig. 7B). The result was consistent with which observed in cancer cell lines. Overall, combination of EpCAM/CD3 BsAb and MUC-1/CD3 BsAb have induced cytokine production in primary tumor cells.

### Combination of EpCAM/CD3 BsAb and MUC-1/CD3 BsAb potently suppressed tumor growth *in vivo*

**Figure 8:**
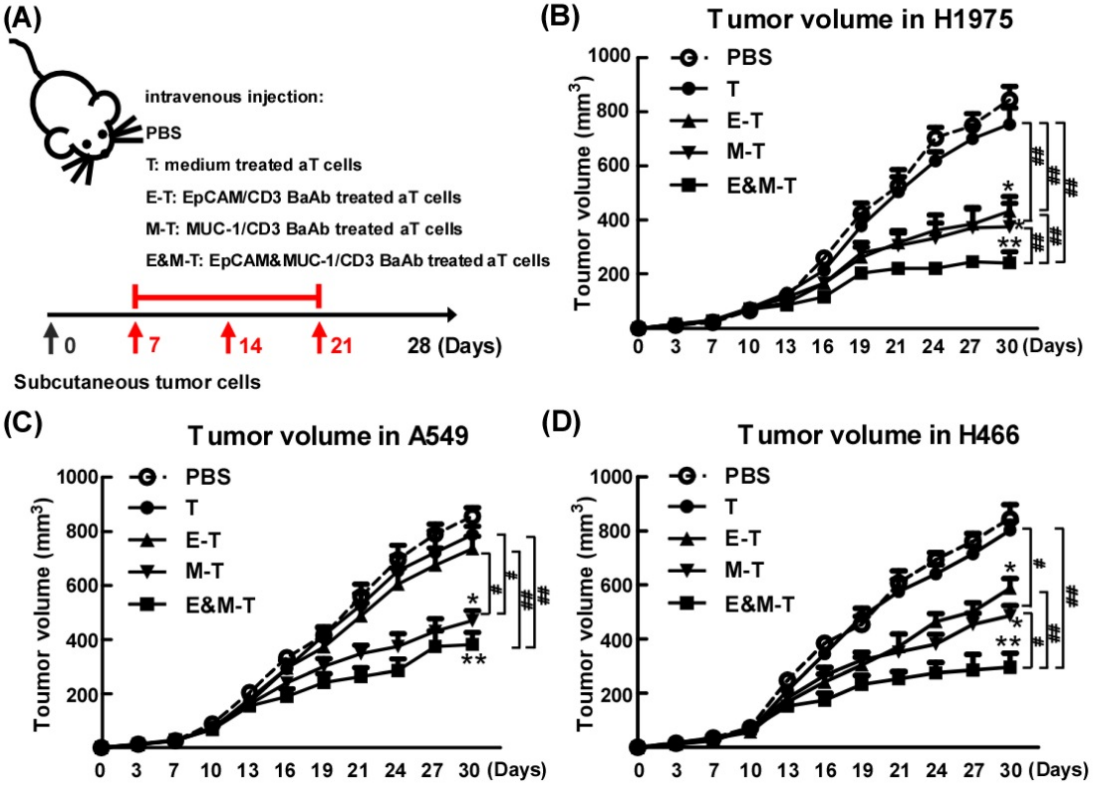
The EpCAM/CD3 BsAb complexed with MUC-1/CD3 BsAb contributes to anti-tumor effects *in vivo*. Tumor-bearing mice were treated with PBS, medium treated aT cells, EpCAM/CD3 BsAb treated aT cells, MUC-1/CD3 BsAb treated aT cells or EpCAM/CD3 BsAb & MUC-1/CD3 BsAb treated aT cells. The treatment plan was indicated in the graphs (A). The H1975 tumor volumes (B), the A549 tumor volumes (C) and the H466 tumor volumes (D) were monitored every 3 days. Bars shown are mean ± SE (n=5), and differences between medium and other groups are determined using one-way ANOVA analysis. *: p < 0.05; **: p < 0.01. Differences between two different groups are statistically different, #: p < 0.05; ##: p < 0.01.

To appraise the effect of combination of EpCAM/CD3 BsAb and MUC-1/CD3 BsAb *in vivo*, tumor bearing mice (H1975) were randomly divided into four groups and received treatment as outlined in the Materials and Methods. When compared with mice treated with PBS, mice on EpCAM/CD3 BsAb, MUC-1/CD3 BsAb or combination of EpCAM/CD3 BsAb and MUC-1/CD3 BsAb significantly reduced tumor volume (Fig. 8B). However, combination of EpCAM/CD3 BsAb and MUC-1/CD3 BsAb were more potent than EpCAM/CD3 BsAb or MUC-1/CD3 BsAb alone to suppress tumor growth in mice (Fig. 8B). Noteworthy, activated T cells by anti-CD3 and anti-CD28 could not induce effective antitumor effect. In addition, we also evaluated the anti-tumor effects of combination of EpCAM/CD3 BsAb and MUC-1/CD3 BsAb on A549 (Fig. 8C) and H466 (Fig. 8D) tumor bearing mice, respectively. The results showed that the effect of EpCAM/CD3 BsAb could not inhibit tumor growth, which was due to the low expression of EpCAM in A549 (Fig. 8C). However, MUC-1/CD3 BsAb alone or combination of EpCAM/CD3 BsAb and MUC-1/CD3 BsAb significantly reduced tumor volume (Fig. 8C). In addition, the therapeutic effect of different BsAbs in H466 tumor bearing mice was similar with H1975 tumor bearing mice (Fig. 8D).

As is known, tumor-draining lymph nodes (TDLNs) were critical site for generating tumor-specific immune responses 21. The regulatory T (Treg) cells in TDLNs were known to suppress immune responses towards tumors, playing a key role in tumor progression and tumor immune escape 22-23. In addition, effectively activated CD8^+^ T cells have resulted in prolonged tumor regression and improved overall response rates in lung cancers 24. Previous studies have shown that increasing T cells in TDLNs was crucial for eliciting effective anti-tumor immune responses and tumor eradication 25. We therefore investigated the effect of different BsAb on T cell population in TDLNs. However, the immunodeficient mice could not be used for further detection of T cell population, so we applied the H1975 tumor bearing BALB/c mice model. The activated T cells (aT cells) were harvested after cultured with anti-mouse CD3 antibody and anti-mouse CD28 antibody followed by IL-2 for 72 h. And then the aT cells were treated with or without EpCAM/CD3 BsAb or MUC-1/CD3 BsAb followed by IL-2 for 72 h to acquire different BsAb treated aT cells (Supplementary methods). Our results showed that as compared with PBS treated H1975 tumor bearing BALB/c mice, the combination of EpCAM/CD3 BsAb and MUC-1/CD3 BsAb reduced the number of Tregs in TDLNs by 15-20% (Fig. S1B) and significantly increased the number of CD8^+^ T cells (Fig. S1C). These results indicated that the function of T cells in TDLNs has been restored after treatment with combination of EpCAM/CD3 BsAb and MUC-1/CD3 BsAb. Although the model of subcutaneous transplantation of H1975 tumor bearing BALB/c mice might not exactly reproduce the immunosuppressive effect of immune microenvironment in lung cancer patients, our results demonstrated that combination of EpCAM/CD3 BsAb and MUC-1/CD3 BsAb could trigger a strong immunotherapeutic and improve immune microenvironment in tumor-bearing mice, which might play an important role in anti-tumor therapy.

## Conclusions

Herein, we described a novel immunotherapy strategy by directly targeting MUC-1 and EpCAM in tumor cells through the combination of EpCAM/CD3 BsAb and MUC-1/CD3 BsAb. The present study showed that combination of EpCAM/CD3 BsAb and MUC-1/CD3 BsAb could dramatically enhance elicited CTL response, as well as promote cytokine production in tumor cell lines and primary tumor cells. More strikingly, combination of EpCAM/CD3 BsAb and MUC-1/CD3 BsAb effectively improved the immune microenvironment by increasing CD8^+^ T cells but decreasing Tregs in TDLNs, thereby led to potent antitumor responses, suggesting its potential advantage for the therapy of targeting EpCAM and MUC-1 on tumor. Hence, combination therapy with EpCAM/CD3 BsAb and MUC-1/CD3 BsAb was expected to be a promising strategy to enhance the anti-tumor efficacy.

## Supplementary Material

Supplementary material and figure S1.

Supplementary video S1.
