# Supplementary material for: The combination therapy with EpCAM/CD3 BsAb and MUC-1/CD3 BsAb elicited antitumor immunity by T-cell adoptive immunotherapy in lung cancer
Source: Int J Med Sci. 2021 Jul 31;18(15):3380–8. doi: 10.7150/ijms.61681 (PMC8436090; doi:10.7150/ijms.61681)
Supplement: Supplementary file 1 — Supplementary material and figure S1. [file ijmsv18p3380s1.pdf]

SUPPLEMENTARY DATA

Methods

The EpCAM/CD3 BsAb and the MUC-1/CD3 BsAb

MUC1/CD3 BsAb or EpCAM/CD3 BsAb were obtained by using 1-Ethyl-3-(3-dimethylaminopropyl) carbodiimide (EDC.HCl) and N-hydroxysuccinimide (NHS) to bind MUC1 antibody or EpCAM antibody and mouse CD3 monoclonal antibody to polylactic acid glycolic acid (PLGA), respectively.

T cells culture, stimulation and activation

The spleens were removed from 5-6 weeks old female healthy BALB/c mice and gently pressed through a nylon mesh to acquire singel cell. And then T cells were sorted from splenocytes by flow cytometry. The activated T cells (aT cells) were harvested after cultured with anti mouse CD3 antibody (1 μg/ml) and anti mouse CD28 antibody (1 μg/ml) followed by IL-2 (100 IU/ml) for 72 h. And then the aT cells were treated with or without EpCAM/CD3 BsAb (10 μg/ml) or MUC-1/CD3 BsAb (10 μg/ml) followed by IL-2 (100 IU/ml) for 72 h, and then washed twice by PBS to acquire the different BsAb treated aT cells (Med: medium, EpCAM: EpCAM/CD3 BsAb, MUC-1: MUC-1/CD3 BsAb and EpCAM & MUC-1: EpCAM/CD3 BsAb & MUC-1/CD3 BsAb).

Tumor implantation and animal immunization

5-6 weeks old female BALB/c mice were subcutaneously (s.c.) injected with H1975 (5×10^6 cells/ mouse) on right buttock. On day 7 after tumor cells implantation, mice were i.v. injected with PBS, activated T cells, or different BsAbs treated activated T cells once a week for 3 weeks. To determine the effect of BsAbs on T cell populations in vivo, tumor-draining lymph nodes (TDLNs) were removed 7 days after last treatment, and cell suspensions were labeled with with PE-anti mouse CD8 to identify $CD8^+$ T cells. To identify Tregs, cell suspensions were first stained with FITC-anti-mouse CD3 and PerCP-anti-mouse CD25 for 30 min, followed by fixation and permeablization with Fix/Perm buffer for another 20 min. The cells were then

labeled with PE-anti-mouse FoxP3, and the percentage of Tregs was determined by measuring $CD3^+$ $CD25^+$ $FoxP3^+$ cells using the flow cytometry.

The dynamically detection of cytotoxic T lymphocyte (CTL) response

The different BsAb treated T cells and target cells (H1975 cells) were cultured in 96-well plates at various effector/target (E: T) ratios for 6 h. H1975 cells were labeled with Dil (2 μg/ml). The different BsAb treated T cells were labeled with Calcein-AM (2μM). T cells and target cells (H1975) were cultured in 24 well plates with effect/target (E: T) ratio of 10:1 for 24 h. CTL response was dynamically monitored by living cell workstation.

Results

Figure S1

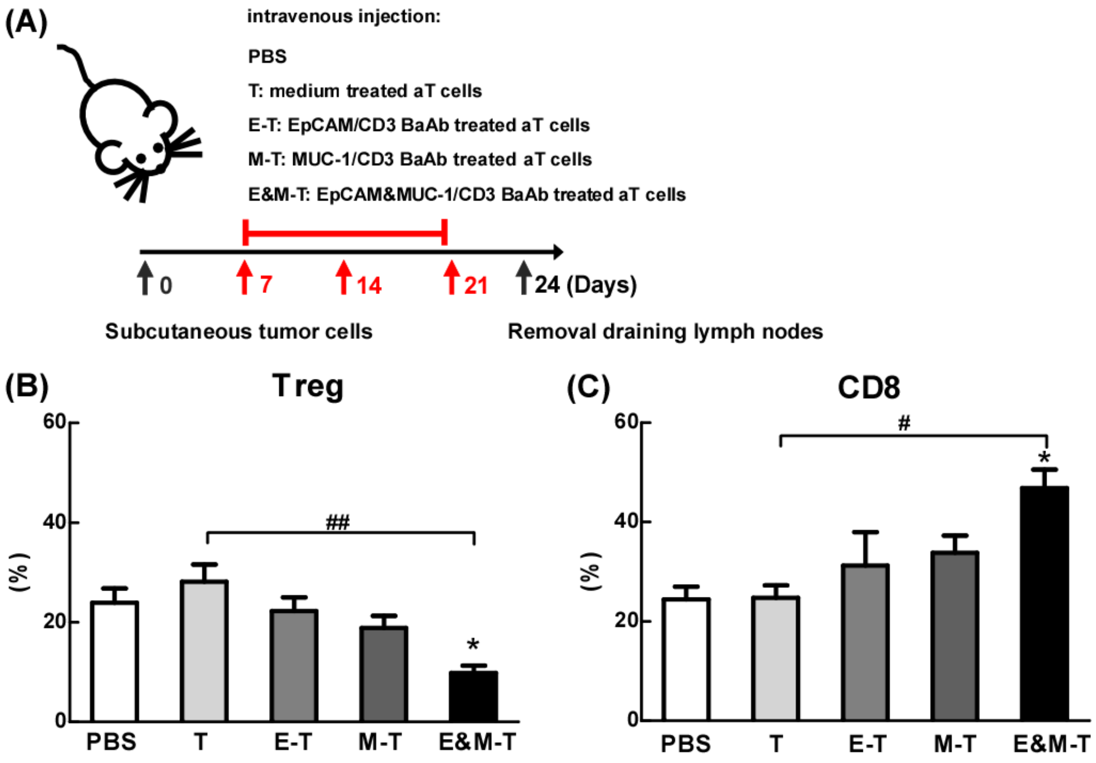

Fig. S1 The effect of different BsAbs on Treg and $CD8^+$ T cells in tumor-draining lymph nodes (TDLNs). BALB/c tumor-bearing mice were immunized with different BsAbs treated T cells as described in materials and methods. The treatment plan was indicated in the graphs (A). On day 3 after 3rd treatment, TDLNs were removed and the percentage of Treg (B, $CD3^+$ $CD25^+$ $FoxP3^+$) and $CD8^+$ T cells (C, $CD8^+$) in

TDLNs were analyzed using flow cytometry. Bars shown are mean ± SE (n=5), and differences between medium and other groups are determined using one-way ANOVA analysis. *: $p < 0.05$. Differences between two different groups are statistically different, #: $p < 0.05$; ##: $p < 0.01$.

Supplementary Video S1

The dynamic detection of CTL by combination of EpCAM/CD3 BsAb and MUC-1/CD3 BsAb in H1975. The results were shown in Supplementary Video S1.
